# Seroprevalence of *Helicobacter pylori* and its association with metabolic syndrome in a rural community of Bangladesh

**DOI:** 10.1002/jgh3.12448

**Published:** 2020-11-24

**Authors:** M. Masudur Rahman, Md. Golam Kibria, Nigar Sultana, Mahfuza Akhter, Hasina Begum, Md. Ahshanul Haque, Rashidul Haque, Shafiqul Alam Sarker, Faruque Ahmed, Mahmud Hasan

**Affiliations:** ^1^ Department of Gastroenterology Sheikh Russel National Gastroliver Institute and Hospital Dhaka Bangladesh; ^2^ Department of Gastroenterology Delta Medical College and Hospital Dhaka Bangladesh; ^3^ Department of Gastroenterology Mughda Medical College and Hospital Dhaka Bangladesh; ^4^ Department of Radiology and Imaging Sheikh Russel National Gastroliver Institute and Hospital Dhaka Bangladesh; ^5^ Nutrition and Clinical Service Division International Centre for Diarrhoeal Diseases Research, Bangladesh (icddr,b) Dhaka Bangladesh; ^6^ Infectious Disease Division International Centre for Diarrhoeal Diseases Research, Bangladesh (icddr,b) Dhaka Bangladesh; ^7^ Gastroliver Foundation Dhaka Bangladesh

**Keywords:** diabetes mellitus, *helicobacter pylori*, metabolic syndrome, non‐alcoholic fatty liver disease, obesity

## Abstract

**Background and Aim:**

As the interrelationship between *Helicobacter pylori*, metabolic syndrome (MetS), and non‐alcoholic fatty liver disease (NAFLD) is controversial, we undertook a community‐based study with the aim to find the seroprevalence of *H. pylori* and its relationship with MetS and NAFLD.

**Methods:**

This door‐to‐door survey was conducted among the adult subjects (≥18 years) of two villages (Charcharia of Dhaka district and Kharrah of Munshiganj district) of Bangladesh. Interviews using a structured questionnaire, clinical examination, anthropometric measurements, ultrasonogram of the liver, and biochemical tests were performed.

**Results:**

Of 1021 subjects, 781 responded (76.49%), and 767 were included in the final analysis (mean age 40.35 ± 15.56 years; female 63.5%). Anti‐*H. pylori* antibodies were found in 418 of 767 (54.5%). There were no *H. pylori* serostatus association with MetS and diabetes mellitus (DM) in univariate or multivariate analysis (all *P* > 0.05). However, *H. pylori* seropositive subjects had lower systolic blood pressure (odds ratio [OR] = −2.95 [−5.58, −0.32]) and low density lipoprotein ‐cholesterol (OR −7.79 [−15, −0.57]) compared to seronegative subjects in the linear regression model. Seronegativity of *H. pylori* was associated with NALFD in univariate (*P* = 0.007) but not multivariate analysis (*P* = 0.086). There were no differences in the frequency of *H. pylori* seropositivity among the participants with nonobese compared to obese NAFLD (19/42 [45%] *vs* 43/99 [43.4%], *P* = 0.844).

**Conclusion:**

In a rural community of Bangladesh, about 55% of the population were *H. pylori* seropositive, which was more common among the underweight than normal or obese subjects. However, there was no relationship observed between *H. pylori* seroprevalence and MetS, DM, or NAFLD.

## Graphical abstract

This door‐to‐door survey was conducted in a rural community of Bangladesh with the aim to find the seroprevalence of *Helicobacter pylori* and its relationship with MetS, metabolic parameters, and NAFLD. About 55% of the population were *H. pylori* seropositive, which was more common among the underweight compared to normal or obese subjects. However, there was no relationship observed between *H. pylori* seroprevalence and the presence of MetS, DM, and NAFLD.

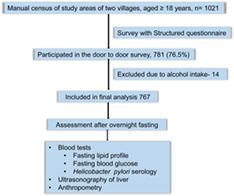

## Introduction

*Helicobacter pylori*, metabolic syndrome (MetS), and non‐alcoholic fatty liver disease (NAFLD) are major public health problems globally. *H. pylori* are the most prevalent chronic bacterial infection of humankind, affecting more than 50% of the world's population.^1^ Although the *H. pylori* prevalence has decreased mainly in developed countries, it is still high in many developing countries.^1^ *H. pylori* infection is primarily associated with gastroduodenal diseases.^2^ However, it may play an important role in various extraintestinal diseases such as MetS and NAFLD.^3^ MetS mainly includes hyperglycemia, hypertension, dyslipidemia, and central obesity.^4^ NAFLD is closely associated with MetS, obesity, and insulin resistance.^5^ The estimated global prevalence of MetS is 20–25%.^6^ The prevalence of MetS was found to be 32.5% in South Asia.^7^ NAFLD is one of the leading causes of chronic liver disease that affects 25.24% of the global population.^8^ The prevalence of NAFLD in Asia is 29.6%.^9^

*H. pylori*, MetS, and NAFLD have common pathogenetic mechanisms such as chronic inflammation, oxidative stress, and counterregulatory hormones.^10^, ^11^ MetS is a cluster of interconnected, co‐occurring metabolic abnormalities that share a common pathophysiological mechanism.^12^ Various inflammatory markers, particularly tumor necrosis factor‐alpha (TNF‐α), interleukin–6 (IL‐6), and C‐reactive protein (CRP), are elevated in MetS.^12^ *H. pylori* infection releases proinflammatory and vasoactive mediators, promotes platelet activation and platelet–leukocyte aggregation, and produces reactive oxygen species.^10^, ^11^ It has recently been reported that *H. pylori* can cause hepatic insulin resistance, which is considered a crucial pathophysiological basis for MetS and NAFLD.^13^ NAFLD is considered the hepatic manifestation of MetS. An international consensus panel has proposed to rename NAFLD as metabolic‐associated fatty liver disease (MAFLD).^14^, ^15^, ^16^ NAFLD has also been found to be associated with gut dysbiosis.^17^ *H. pylori* infection causes changes in the gut microbiota.^18^, ^19^ Obesity and other metabolic syndrome parameters are related to alterations of gut microbiota.^20^, ^21^ Hence, there may be a possible connection between *H. pylori* infection, NAFLD, and MetS or components of the MetS.

The association of *H. pylori* with MetS and NAFLD is controversial. Nevertheless, such an association is plausible, particularly in areas where the prevalence of *H. pylori*, MetS, and NAFLD is high. Although the seroprevalence among the general adult population is currently unknown, selected population‐based studies in Bangladesh reported high seroprevalences of *H. pylori*.^22^, ^23^ The prevalence of MetS and NAFLD was also found to be high in Bangladesh. A recent systematic review of the prevalence of MetS in Bangladesh found that 37% of the population had MetS.^24^ The prevalence of NAFLD was found to be 34% in population‐based studies in Bangladesh.^25^ Establishing an association between *H. pylori*, MetS, and NAFLD has important clinical implications. If such an association is proved, *H. pylori* eradication may have a beneficial effect on MetS‐ and NAFLD‐related morbidity and mortality. Therefore, we have conducted a study among a rural community of Bangladesh with the aims to : (i) estimate the seroprevalence of *H. pylori*, (ii) find the association between *H. pylori* and MetS, (iii) observe the association between *H. pylori* and the factors associated with MetS, and (iv) find the association between *H. pylori* and NAFLD.

## Methods

### *Study design and study population*

**Figure 1:**
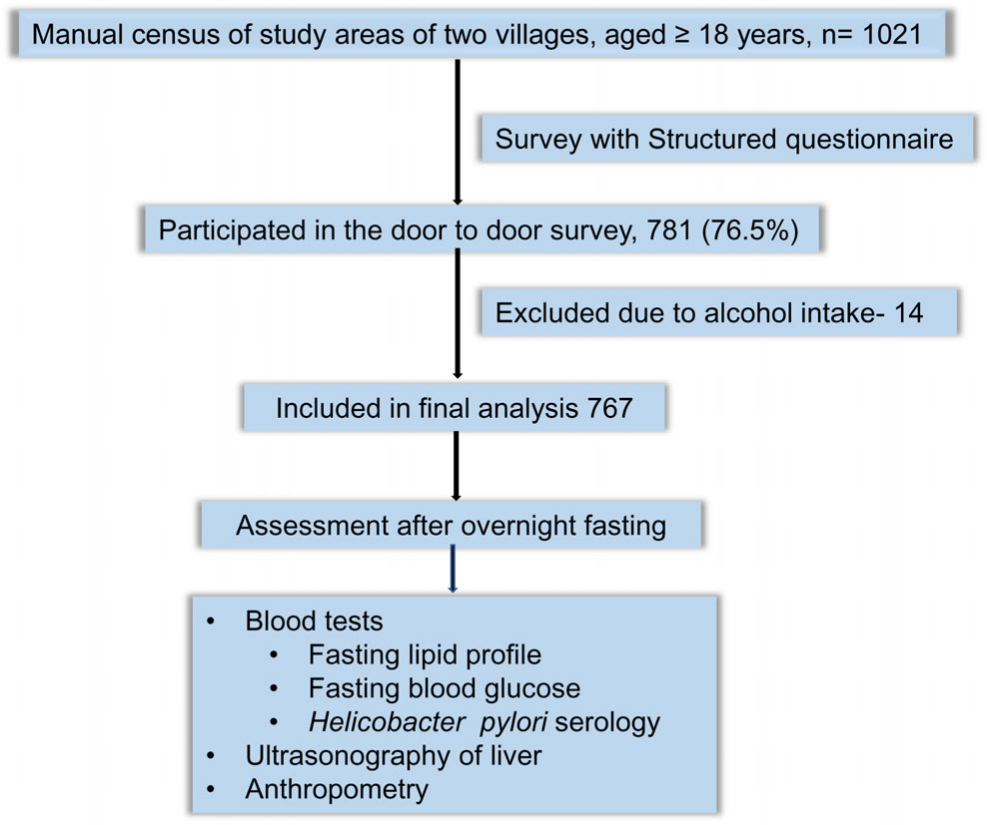
Study outline.

This was a cross‐sectional study with a house‐to‐house survey conducted among the adult population (≥18 years) of two villages, namely, Charcharia of Nawabganj upazila of Dhaka district and Kharrah of Srinagar upazila of Munshiganj district, in Bangladesh from April 2014 to February 2015. Three trained research assistants (RAs) conducted the interviews using a structured questionnaire and anthropometric measurements. The investigators performed clinical examinations. Ultrasonography of the hepatobiliary system and biochemical tests were carried out after overnight fasting. Figure 1 shows the study outline. One investigator entered the data, 10% of which were cross‐checked by another investigator. The study protocol was approved by the Institutional Ethics Committee, and written informed consent was obtained from the participants.

### *Questionnaire*

A structured questionnaire was used during interviews with the participants. The questionnaire had two subsections: (i) sociodemographic information and (ii) history of chronic disease and history of medication use. Sociodemographic data included age, gender, occupation, monthly family income, education, marital status, religion, smoking, and alcohol intake. History of chronic disease included DM, hypertension, dyslipidemia, chronic hepatitis B and C infections, autoimmune hepatitis, primary biliary cholangitis, and drug‐induced liver injury. Medication history also included drugs taken for DM, hypertension, and dyslipidemia.

### *Clinical examinations and anthropometric measurement*

The trained RAs took anthropometric measurements that included height (cm), weight (kg), and waist circumference (cm). The investigators performed the clinical examinations to assess the presence of stigmata of chronic liver disease. Pulse, systolic (mm Hg) and diastolic (mm Hg) blood pressure (BP) were measured in the resting position and were recorded.

### *Biochemical tests*

Venous blood samples were collected from fasting subjects using sterile disposable syringes and needles in an aseptic manner. Blood samples were centrifuged (4000 rpm), and serum samples were collected and stored at −20°C until assay for serum glucose, triglyceride (TG), total cholesterol (TCh), high‐density lipoprotein cholesterol (HDL‐C), and low‐density lipoprotein cholesterol (LDL‐C) using an automatic biochemistry analyzer (Roche, Rotkreuz, Switzerland). Serum samples were also tested for Immunoglobulin G (IgG) antibody responses to an *H. pylori* membrane protein (MP) antigen (Hel 305 MP) using an enzyme‐linked immunosorbent assay (ELISA). A commercially available ELISA kit was used following the manufacturer's (Human, Wiesbaden, Germany) instructions.

### *Abdominal ultrasonography*

Abdominal ultrasonography (Wuxi Haiying International Trade Co. Ltd., Wuxi, China) was performed by a senior radiologist trained in ultrasound on fasting patients in a community health service center to detect NAFLD. Fatty liver was diagnosed in the presence of two of the three following criteria: (i) increased hepatic echogenicity compared to the spleen or the kidney, (ii) blurring of liver vasculature, and (iii) deep attenuation of the ultrasonographic signal.^26^

### *Definitions*

MetS was defined as per the Revised National Cholesterol Education Program, Adult treatment panel III (revised NCEP ATP III), which required the presence of three or more of the following features: (i) waist circumference ≥ 90 cm in men or ≥ 80 cm in women; (ii) TG level of 150 mg/dL or higher; (iii) HDL‐C level less than 40 mg/dL in men and less than 50 mg/dL in women; (iv) systolic blood pressure (SBP) of 130 mm Hg or higher or diastolic blood pressure (DBP) of 85 mm Hg or higher; and (v) fasting plasma glucose level of 110 mg/dL or higher.^27^ Obesity was defined as per the World Health Organization (WHO) criteria. Body mass index (BMI) ≥25, 25.0–29.99, and >30 kg/m^2^ were defined as overweight, preobese, and obese, respectively.^28^ Lean NAFLD and nonobese NAFLD were defined by BMI <23 kg/m^2^ and <25 kg/m^2^, respectively.^29^ Diabetes mellitus (DM) was diagnosed if the fasting blood glucose (FBG) value was ≥7.0 mmol/L or the subject already had medications for DM. Impaired fasting glucose (IFG) was diagnosed if the FBG was between ≥6.1 and <7.0 mmol/L.^30^

### *Statistical analysis*

All statistical analyses were performed using STATA (Stata Statistical Software: Release 13. College Station, TX, USA: StataCorp LP). Descriptive statistics, such as proportion for categorical variables and mean and standard deviation for quantitative variables, were used to summarize the data. Chi‐square and proportion tests were used to observe the association between two categorical variables, and a *t*‐test was used to find the mean difference between two groups of a normally distributed continuous variable. A linear regression model was used after adjusting for age, gender, religion, marital status, smoking habit, education, and occupation to observe the adjusted mean difference of parameters of MetS between *H. pylori* serology‐positive and ‐negative subjects. Multiple logistic regression was used to assess the association between *H. pylori* serology and gender, religion, BMI, DM, marital status, smoking, occupation, education, MetS, and NAFLD.

## Results

Of 1021 subjects, 781 (76.49%) responded, and 767 were included in the final analysis after excluding 14 subjects who had a history of any amount of alcohol consumption (Fig. 1). Of them, 495 were female (64.5%). The mean age of the study subjects was 40.35 ± 15.56 years.

### *Seroprevalence of H. pylori*

**Figure 2:**
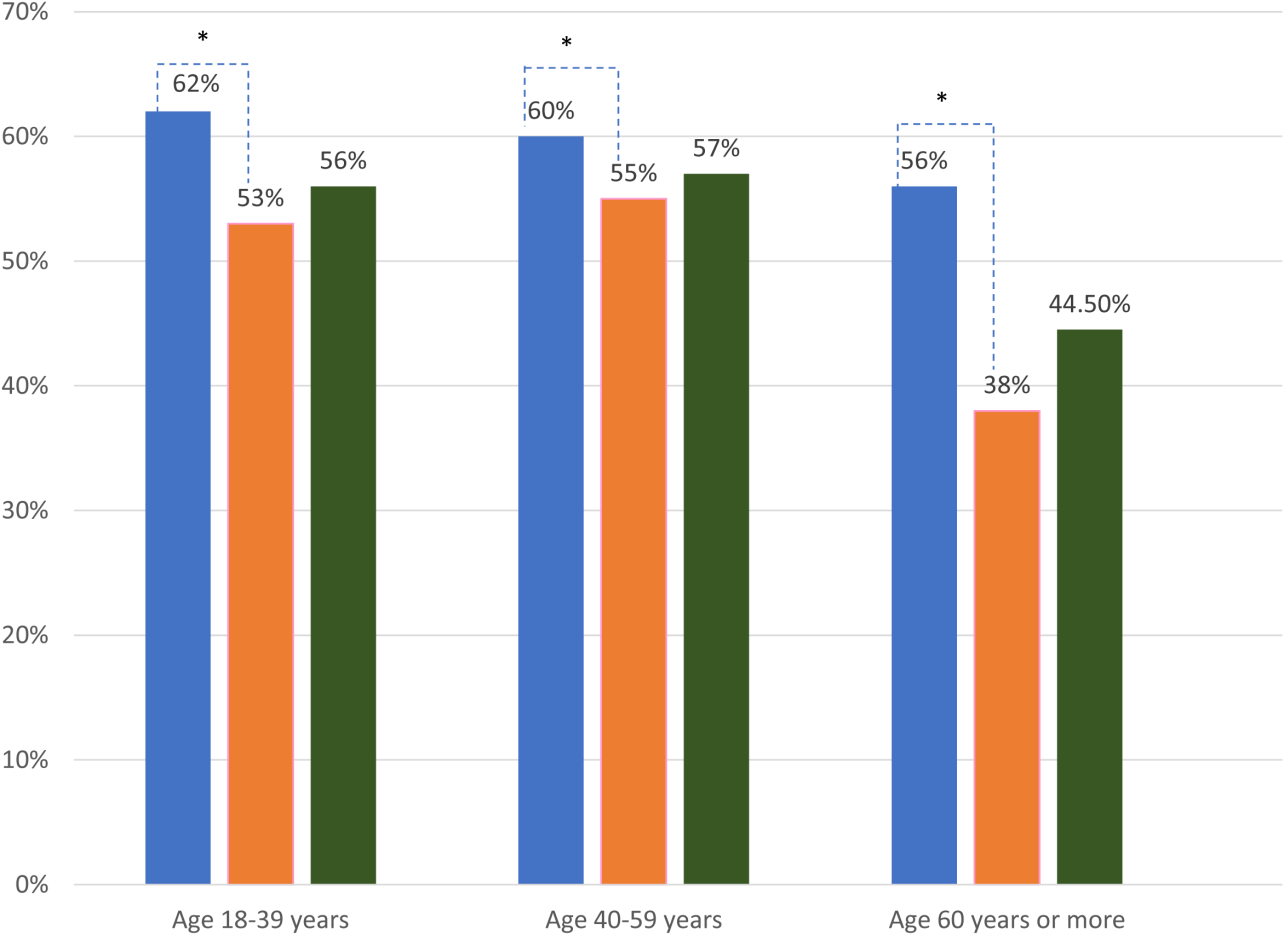
Age‐ and gender‐specific prevalence of *Helicobacter pylori*. *Pvalue > 0.05. (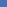), Male; (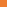), female; (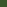), overall.

Overall, positive IgG‐specific anti‐*H. pylori* antibodies in serum were found in 418 of 767 (54.5%) participants. There was no difference in age between subjects with and without *H. pylori* (mean age 39.77 ± 14.65 years *vs* mean age 40.97 ± 16.48; *P* = 0.25). Age‐ and gender‐specific seroprevalence of *H. pylori* is shown in Figure 2.

### *Sociodemographic factors associated with H. pylori seropositivity*

Table 1 shows the sociodemographic characteristics and presence of MetS and NAFLD of the subjects with and without *H. pylori* seropositivity. The prevalence of seropositivity among males was higher than females (59.6 *vs* 51.7%, *P* = 0.037). The prevalence of seropositivity of *H. pylori* was more common among married individuals compared to a single person (57.4 *vs* 42.2%, *P* = 0.001), cultivators compared to homemakers and other occupations (54 *vs* 52.2%, *P* = 0.038), and smokers compared to nonsmokers (63.2 *vs* 52.7%, *P* = 0.039).

**Table 1:** Sociodemographic characteristics, metabolic syndrome, and non‐alcoholic fatty liver disease among *Helicobacter pylori* seropositive and seronegative subjects

Characteristics, *n* (%)	*H. pylori* seropositive (*n* = 418)	*H. pylori* seronegative (*n* = 349)	*P‐*value
Age (mean ± SD)	39.76 ± 14.70	41.06 ± 16.54	0.255
Gender			
Male	162 (38.8)	110 (31.5)	0.041
Female	256 (61.2)	239 (68.5)	
Marital status			
Married	356 (85.2)	264 (75.6)	<0.001
Single	62 (14.8)	85 (24.4)	
Occupation			
Housewife	241 (57.7)	221 (63.3)	0.038
Cultivator and day laborer	68 (16.3)	35 (10.0)	
Service‐holder and others	104 (26.1)	93 (26.6)	
Education			
Up to Class IV	201 (48.1)	158 (45.2)	0.118
Classes V–X	189 (45.2)	153 (43.8)	
>X class	28 (6.7)	38 (10.9)	
Monthly Income			
≤10 000 TK	251 (61.4)	206 (59.5)	0.608
>10 000 TK	158 (38.3)	140 (40.5)	
Smoking Status			
Smoker (Current or Past)	74 (14.1)	46 (13.3)	0.039
Nonsmoker	334 (80.9)	300 (86.7)	
Religion			
Muslim	335 (80.1)	283 (81.1)	0.784
Hindu	83 (19.9)	66 (18.9)	
Presence of MetS	126 (30.1)	128 (36.7)	0.064
Weight Status			
Underweight	79 (18.9)	40 (11.5)	0.003
Normal weight	221 (52.9)	173 (49.6)	
Overweight	92 (22)	104 (24.8)	
Obese	26 (6.25)	32 (9.2)	
BMI (mean ± SD)	22.70 ± 4.45	23.85 ± 4.45	<0.001
Presence of NAFLD	62 (14.8)	79 (22.6)	0.007
Presence of DM	47 (11.2)	48 (13.8)	0.322

BMI, body mass index; DM, diabetes mellitus; MetS, metabolic syndrome; NAFLD, non‐alcoholic fatty liver disease; SD, standard deviation.

### *H. pylori serostatus and obesity and metabolic syndrome*

*H. pylori* seropositivity was more common among participants with underweight (66.4%) compared to those with normal weight (56.1%) and overweight (46.9%) and obesity (44.8%) (*P* = 0.003). The mean BMI of the *H. pylori* seropositive persons was lower than the seronegative person (22.7 ± 4.45 kg/m^2^ *vs* 23.85 ± 4.45 kg/m^2^, *P* < 0.001). Overall, MetS was present in 254 (33.1%) participants. There were no differences in the frequencies of DM or MetS between *H. pylori* seropositive and seronegative subjects, as shown in Table 1.

### *H. pylori serostatus and* *NAFLD*

NAFLD was present in 141 (18.4%) participants. The frequency of NAFLD was lower among the *H. pylori* seropositive subjects compared to the seronegative subjects (14.8 *vs* 22.6%), as shown in Table 1. Among the NAFLD subjects, 42 (29.8%) were nonobese, 99 (70.2%) were obese, 19 (13.5%) were lean, and 122 (86.5%) were nonlean. There were no differences in the frequency of *H. pylori* seropositivity among the participants with nonobese compared to obese NAFLD (19/42 [45%] *vs* 43/99 [43.4%], *P* = 0.844) and lean compared to nonlean NAFLD (9/19 [47.4%] *vs* 53/122 [43.4%], *P =* 0.784).

### *Multivariate analysis for risk factors of H. pylori*

On multivariate analysis, the presence of underweight and being married was found to be the risk factor for *H. pylori* seropositivity, as shown in Table 2.

**Table 2:** Multivariate analysis of risk factors of *Helicobacter pylori* seropositivity

	Unadjusted OR (95% CI)	*P*‐value	Adjusted OR (95% CI)	*P*‐value
Age	0.99 (0.98, 1.00)	0.25	0.99 (0.98, 1.00)	0.072
Gender				
Male	Reference		Reference	
Female	0.73 (0.54, 0.98)	0.037	0.80 (0.46, 1.41)	0.443
Religion				
Islam			Reference	
Hindu	1.06 (0.74, 1.52)	0.742	0.97 (0.66, 1.43)	0.886
BMI				
Underweight	Reference		Reference	
Normal	0.65 (0.42, 0.99)	0.047	0.60 (0.38, 0.95)	0.030
Overweight	0.45 (0.28, 0.72)	0.001	0.43 (0.25, 0.73)	0.002
Obese	0.41 (0.22, 0.78)	0.007	0.43 (0.20, 0.89)	0.023
DM				
Present	Reference		Reference	
Absent	1.26 (0.82, 1.94)	0.294	0.98 (0.58, 1.66)	0.953
Marital status				
Married	Reference		Reference	
Single	0.54 (0.38, 0.78)	0.001	0.48 (0.31, 0.72)	0.000
Smoking				
Smoker	Reference		Reference	
Nonsmoker	0.65 (0.44, 0.96)	0.032	0.82 (0.51, 1.31)	0.404
Occupation				
Housewife	Reference		Reference	
Cultivator and day laborer	1.78 (1.14, 2.79)	0.011	1.45 (0.78, 2.68)	0.239
Service‐holder and others	1.07 (0.77, 1.50)	0.67	1.06 (0.58, 1.91)	0.856
Monthly income				
≤10 000 TK	Reference		Reference	
>10 000 TK	0.93 (0.69, 1.24)		1.01 (0.74, 1.39)	0.933
Metabolic syndrome				
Present	Reference		Reference	
Absent	1.34 (0.99, 1.81)	0.056	0.90 (0.61, 1.34)	0.614
NAFLD				
Present	Reference		Reference	
Absent	1.68 (1.16, 2.43)	0.006	1.50 (0.94, 2.39)	0.088
Education in three groups				
Illiterate to class IV	Reference		Reference	
Class V to class X	0.97 (0.72, 1.31)	0.847	0.88 (0.62, 1.26)	0.489
More than class X	0.58 (0.34, 0.98)	0.044	0.53 (0.29, 0.99)	0.046

*Adjusted for all variables included in the multivariable model.

BMI, body mass index; CI, confidence interval; NAFLD, non‐alcoholic fatty liver disease; OR, odds ratio.

### *H. pylori serostatus and parameters of* *MetS*

Table 3 shows the comparison of *H. pylori* seropositive with seronegative subjects regarding waist circumference, systolic blood pressure (SBP), diastolic blood pressure (DBP), FBG, fasting TCh, HDL‐C, LDL‐C, and TG. Mean SBP and serum LDL‐C were significantly lower among subjects with *H. pylori* seropositivity on univariate analysis (Table 3). After adjusting for age, gender, religion, marital status, smoking status, education, and occupation in the linear regression model, SBP and LDL‐C were 2.95 mm Hg and 7.79 mg/dL lower, respectively, among *H. pylori* seropositive subjects compared to seronegative subjects (Table 4).

**Table 3:** *Helicobacter pylori* serostatus and parameters of metabolic syndrome

Characteristics	*H. pylori* seropositive (*n* = 418)	*H. pylori* seronegative (*n* = 349)	*P‐*value
Waist circumference	77.24 ± 31.29	78.85 ± 11.64	0.364
Systolic blood pressure	112.90 ± 19.75	116.67 ± 19.02	0.008
Diastolic blood pressure	75.94 ± 11.58	77.52 ± 11.31	0.057
Fasting blood glucose	5.46 ± 2.17	5.47 ± 5.47	0.939
Serum total cholesterol	185.44 ± 44.65	191.76 ± 72.25	0.139
Serum HDL cholesterol	40.41 ± 10.84	41.11 ± 12.37	0.407
Serum LDL cholesterol	116.0 ± 35.41	123.78 ± 62.76	0.033
Serum Triglycerides	148.01 ± 87.19	154.40 ± 123.71	0.121

HDL, high‐density lipoprotein; LDL, low‐density lipoprotein.

**Table 4:** Effects of *H. pylori* serostatus on metabolic syndrome‐related variables

Metabolic parameters	Unadjusted coefficient (95% CI)	*P*‐value	Adjusted coefficient (95% CI)*	*P*‐value
Systolic blood pressure	−3.77 (−6.53, −1.00)	0.008	−2.95 (−5.58, −0.32)	0.028
Diastolic blood pressure	−1.58 (−3.21, 0.05)	0.057	−1.35 (−2.99, 0.29)	0.106
Fasting blood glucose	−0.01 (−0.31, 0.28)	0.939	0.02 (−0.27, 0.32)	0.881
Total cholesterol	−6.32 (−14.7, 2.06)	0.139	−5.56 (−13.92, 2.8)	0.192
Serum LDL Cholesterol	−7.69 (−14.78, −0.6)	0.033	−7.79 (−15, −0.57)	0.034
Serum HDL Cholesterol	−0.70 (−2.35, 0.95)	0.407	−0.73 (−2.38, 0.92)	0.384
Serum triglycerides	−11.89 (−26.91, 3.14)	0.121	−12.47 (−27.81, 2.88)	0.111
Waist circumference	−1.61 (−5.09, 1.87)	0.364	−0.37 (−3.89, 3.15)	0.837

*Adjusted for age, gender, religion, marital status, smoking status, education, and occupation in the linear regression model. Dependent variable: metabolic syndrome indicators; independent variable*: Helicobacter pylori* serology; reference category is negative.

CI, confidence interval; HDL, high density lipoprotein; LDL, low density lipoprotein.

## Discussion

This cross‐sectional study was conducted in a population living in a rural community in Bangladesh. High seroprevalence (54.5%) of *H. pylori* among the adult population was observed. *H. pylori* seropositivity was more common among the underweight than normal or overweight‐obese subjects. The *H. pylori* serostatus was not associated with MetS, NAFLD, or DM. However, after adjusting for age, gender, religion, marital status, smoking status, education, and occupation in the linear regression model, *H. pylori* seropositive subjects had significantly lower SBP and serum LDL‐C compared to seronegative subjects.

The prevalence of *H. pylori* infection among the selected population has been reported to be high in Bangladesh. A urea breath test‐based study among the young population has demonstrated that 84% of children become infected with *H. pylori* by 6–9 years in Bangladesh.^23^ Another pilot study conducted in 1995 reported 92% seropositivity of *H. pylori* among the asymptomatic adult overseas job seekers.^22^ A study, conducted two decades later, on similar overseas job seekers reported 71% seropositivity for *H. pylori* infection.^31^ Although there was a methodological limitation with small sample size, those studies nevertheless demonstrated that the seroprevalence was high among the asymptomatic adult population in Bangladesh and highlighted the need for a large‐scale community‐based study. This study addressed the issues and surveyed a community with a larger population group, which we believe to be a representative sample from Bangladesh's perspective. Compared to those previous studies, the present study demonstrated a lower rate of seropositivity in the adult population. Improved health awareness, personal hygiene, and socioeconomic or educational status over the past few years^32^ or widespread use of the antimicrobial drugs in Bangladesh could be the reason for this lower seropositivity observed in the present study. This finding of the current seroprevalence of *H. pylori* is consistent with a recent systematic review and meta‐analysis on the global prevalence of *H. pylori*, which found that the prevalence varied from 43.1 to 79.5% in Asia with a prevalence rate of 61.6% in Southern Asia.^1^

MetS is a cluster of metabolic abnormalities for which insulin resistance (IR) plays a pivotal role.^12^ The potential association of *H. pylori* infection with MetS and its components, including obesity, dyslipidemia, DM, and NAFLD, is controversial. There are several plausible mechanisms of such associations. *H. pylori* infection causes hepatic insulin resistance through the c‐jun/suppressor of cytokine signaling (SOCS) 3 pathway.^13^ *H. pylori* releases proinflammatory and vasoactive mediators like IL‐1, IL‐6, IL‐8, IL‐10, IL‐12, TNF‐α, interferon ϒ, leukotrienes, and prostaglandins and acute phase reactants like fibrinogen and CRP, which are also involved in the pathogenesis of IR. *H. pylori* infection promotes platelet activation and aggregation, which may also play a role in IR. In addition, *H. pylori* infection produces reactive oxygen species involved in the pathogenesis of IR syndrome.^10^, ^11^, ^13^, ^33^

The present study did not find any association of *H. pylori* seropositivity and MetS. Similar findings were reported by Naja *et al*., who did not find any association of *H. pylori* infection with IR and MetS among the Lebanese adults.^34^ Although a meta‐analysis with significant heterogeneity found an association between *H. pylori* and MetS,^35^ an inverse relationship was found between CagA‐positive *H. pylori* infection and fatal cardiovascular events, according to a population‐based cohort study in Germany.^36^ High frequency of *H. pylori‐*associated virulence factors, particularly in the present study population, has been reported earlier.^37^

Association of *H. pylori* with NAFLD remains controversial as some of the epidemiological studies found an association,^38^, ^39^ whereas others did not.^40^, ^41^ Possible mechanisms of pathogenesis of NAFLD induced by *H. pylori* include (i) insulin resistance; (ii) inflammatory cytokines or adipokines, particularly CRP, TNF‐α, and IL‐6; (iii) altered lipid profile; and (iv) altered intestinal permeability and gut microbiota.^42^ *H. pylori* seronegativity was associated with NAFLD in univariate analysis but not in multivariate analysis in the present study. A few recent meta‐analyses of observational studies suggest a positive *association* between *H. pylori* and NAFLD.^5^, ^43^, ^44^ However, there are concerns due to moderate to high heterogenicity and significant publication bias in those studies.^5^

No relationship was found between *H. pylori* infection and DM, and this finding corroborated sufficiently with previous findings.^45^, ^46^ Although a meta‐analysis of case–control studies found a significant association between *H. pylori* and DM, the authors acknowledged significant heterogenicity.^47^ Therefore, the association between *H. pylori* infection and DM remains inconclusive.

Some epidemiological studies found an association of *H. pylori* infection with obesity,^48^, ^49^ whereas others have failed to show such an association.^50^, ^51^ In the present study, *H. pylori* infection was associated with underweight rather than obesity. In a case–control study in Taiwan, *H. pylori* were more common among normal or underweight subjects than in obese subjects.^52^ The low BMI or underweight among *H. pylori*‐infected patients may result from persistent gastric inflammation, resulting in the dysregulation of appetite and calorie homeostasis, mediated by the gut hormone ghrelin. It has been shown that childhood acquisition of *H. pylori* infection may cause decreased appetite and low BMI.^53^, ^54^ *H. pylori*‐associated dyspepsia may further deteriorate nutritional status in infected subjects, without such an infection occurring in adult life. Moreover, it has been found that, after eradication, *H. pylori*‐infected patients gained weight.^55^, ^56^ Most of the *H. pylori* infection occurs in childhood in Bangladesh.^23^ Such *H. pylori* acquisition in childhood may result in low BMI among the seropositive subjects in adult life. A recent study from Bangladesh found an association between *H. pylori* infection and fecal biomarkers of environmental enteric dysfunction among children. These findings suggest that the acquisition and persistence of *H*. *pylori* infection in the early years of life may exert an adverse impact on intestinal health and induce gut inflammation, resulting in increased intestinal permeability, and may cause malnutrition in adult life.^57^

Gut microbiota has been found to play an essential role in the pathogenesis of obesity.^20^, ^21^ Dysregulated production of inflammatory adipokines caused by excess or dysfunction of adipose tissue can contribute to the development of IR and obesity‐related metabolic diseases, including NAFLD.^58^ Alterations of gut microbiota or dysbiosis have also been demonstrated in *H. pylori* infection.^18^, ^19^ The present study indicates more common seropositivity among underweight populations, therefore, suggesting a need for further study elucidating relationship betwee *H. pylori* and dysbiosis.

In the present study, *H. pylori* seropositivity was associated with low LDL‐C and low SPB. Such a relationship may result from low BMI and underweight among the *H. pylori* seropositive subjects. The effects of eradication of *H. pylori* on lipid profiles have been found to be contradictory. An open‐label study among dyspeptic patients found a significant decrease in the serum levels of total cholesterol and LDL‐C 3 months after *H. pylori* eradication.^59^ On the other hand, a case–control study found that eradication of *H. pylori* significantly increases the incidence of hyperlipidemia and obesity in patients with a peptic ulcer.^56^ In another study, eradication of *H. pylori* was associated with a significant increase in total cholesterol, TGs, and HDL‐C.^60^

The inconsistent findings of the association between *H. pylori* and MetS and its components, including DM, NAFLD, obesity, and dyslipidemia, may be attributed to few factors. First, there are differences in study populations, such as diseased patients, elderly subjects, healthy volunteers, or the general population. Second, there are concerns about the methodology used to diagnose *H. pylori* infection, such as serology or urea breath test. Third, there are differences in the definition of MetS used in different studies. Fourth, there are differences in the BMI cutoff points used to define weight status. Fifth, there are differences in study settings, such as hospital *versus* community. Finally, there are variations of the degree of adjustment for potential confounders. Such adjustment is essential as the pathogenesis of MetS and its component parameters are multifactorial, which includes genetic susceptibility, environmental factors, immune response, personal habit, and phenotypic expression.^61^

One of the present study's limitations is that it was conducted in a selected area in Bangladesh. As Bangladesh is a small country, and 80% of the population lives in rural areas, the seroprevalence of *H. pylori* as observed may represent the majority of the population of the country. Moreover, this study attempts to find an association between *H. pylori* and MetS and NAFLD in a rural South Asian population. Such data from a rural region is notably less or unreported. Another limitation may be that the dietary assessment of the study participants was not investigated in this study. To the best of our knowledge, this is the first study demonstrating about half of the population being seropositive for *H. pylori* infection in Bangladesh. This finding has important implications for selecting the appropriate management strategies for *H. pylori i*nfection.

In conclusion, although just above half of the rural community population is seropositive to *H. pylori* infection, it is not associated with MetS, including DM and NAFLD in Bangladesh.
